# Aging immunity: unraveling the complex nexus of diet, gut microbiome, and immune function

**DOI:** 10.1097/IN9.0000000000000061

**Published:** 2025-05-09

**Authors:** Khatereh Babakhani, Amanda L. Kucinskas, Xiangcang Ye, Erin D. Giles, Yuxiang Sun

**Affiliations:** 1 Department of Nutrition, Texas A&M University, College Station, TX, USA; 2 Department of Molecular & Integrative Physiology, University of Michigan, Ann Arbor, MI, USA; 3 School of Kinesiology, University of Michigan, Ann Arbor, MI, USA

**Keywords:** aging, gut microbiome, diet, immune system, fiber, fat, protein, omega-3 fatty acids, tryptophan, galacto-oligosaccharides, high-fat diet, high-fiber diet, high-protein diet

## Abstract

Aging is associated with immune senescence and gut dysbiosis, both of which are heavily influenced by the diet. In this review, we summarize current knowledge regarding the impact of diets high in fiber, protein, or fat, as well as different dietary components (tryptophan, omega-3 fatty acids, and galacto-oligosaccharides) on the immune system and the gut microbiome in aging. Additionally, this review discusses how aging alters tryptophan metabolism, contributing to changes in immune function and the gut microbiome. Understanding the relationship between diet, the gut microbiome, and immune function in the context of aging is critical to formulate sound dietary recommendations for older individuals, and these personalized nutritional practices will ultimately improve the health and longevity of the elderly.

## 1. Introduction

The number of adults aged 65 years or older in the United States is projected to grow from 58 million (17% of the population) in 2022 to 82 million (23% of the population) by 2050 ^[1]^. Aging is associated with a gradual decline of the immune system (immunosenescence) and the progressive development of low-grade systemic inflammation (inflammaging). Immunosenescence and inflammaging lead to reduced vaccine efficacy, elevated susceptibility to infections, and increased risk of chronic inflammatory diseases, all of which contribute to increased morbidity and health care burden ^[2,3]^. Therefore, novel strategies to mitigate immunosenescence and inflammaging are critically needed given the fast-growing aging populations.

One potential strategy for targeting immunosenescence and inflammaging is through the modifiable risk factor of diet. Diet composition and individual nutrients have been shown to profoundly impact the cellular makeup and function of the immune system ^[4,5]^. Additionally, essential nutrients from the diet have been shown to modulate the composition of the gut microbiome, some of which can exert anti-inflammatory and immune-supportive effects ^[6,7]^. Given these intriguing functional connections, understanding how different dietary components affect the immune system and gut microbiota during aging is of great importance. Here, we aim to review the current data on how innate and adaptive immunity, inflammatory responses, and the gut microbiome are reprogrammed during aging. We specifically highlight the influence of high-fiber, high-protein, and high-fat diets, as well as individual dietary components, such as tryptophan, omega-3 fatty acids, and galacto-oligosaccharides, in the context of aging.

## 2. The innate immune system in aging

The innate immune system is essential in the rapid mobilization of immune cells, including monocytes, macrophages, dendritic cells, neutrophils, and natural killer cells, to the sites of infection and inflammation, where they contribute to the production of inflammatory cytokines ^[8]^. Aging is associated with pronounced dysfunctions in the innate immune system. For example, aging leads to reduced macrophage responses, including reduced tissue infiltration, phagocytosis, chemokine/cytokine secretion, slowed wound healing, and impaired antigen presentation ^[9]^. Similarly, the effectiveness of neutrophils also decreases, limiting their ability to phagocytose, produce reactive oxygen intermediary molecules, and eradicate intracellular substances ^[10]^. In this section, we highlight the impact of aging on the cells of the innate immune system, specifically focusing on the effects on specific cell subsets, cytokine production, and cluster of differentiation (CD) marker molecules from studies in both humans and rodent models.

### 2.1 Monocytes

**Figure 1.**
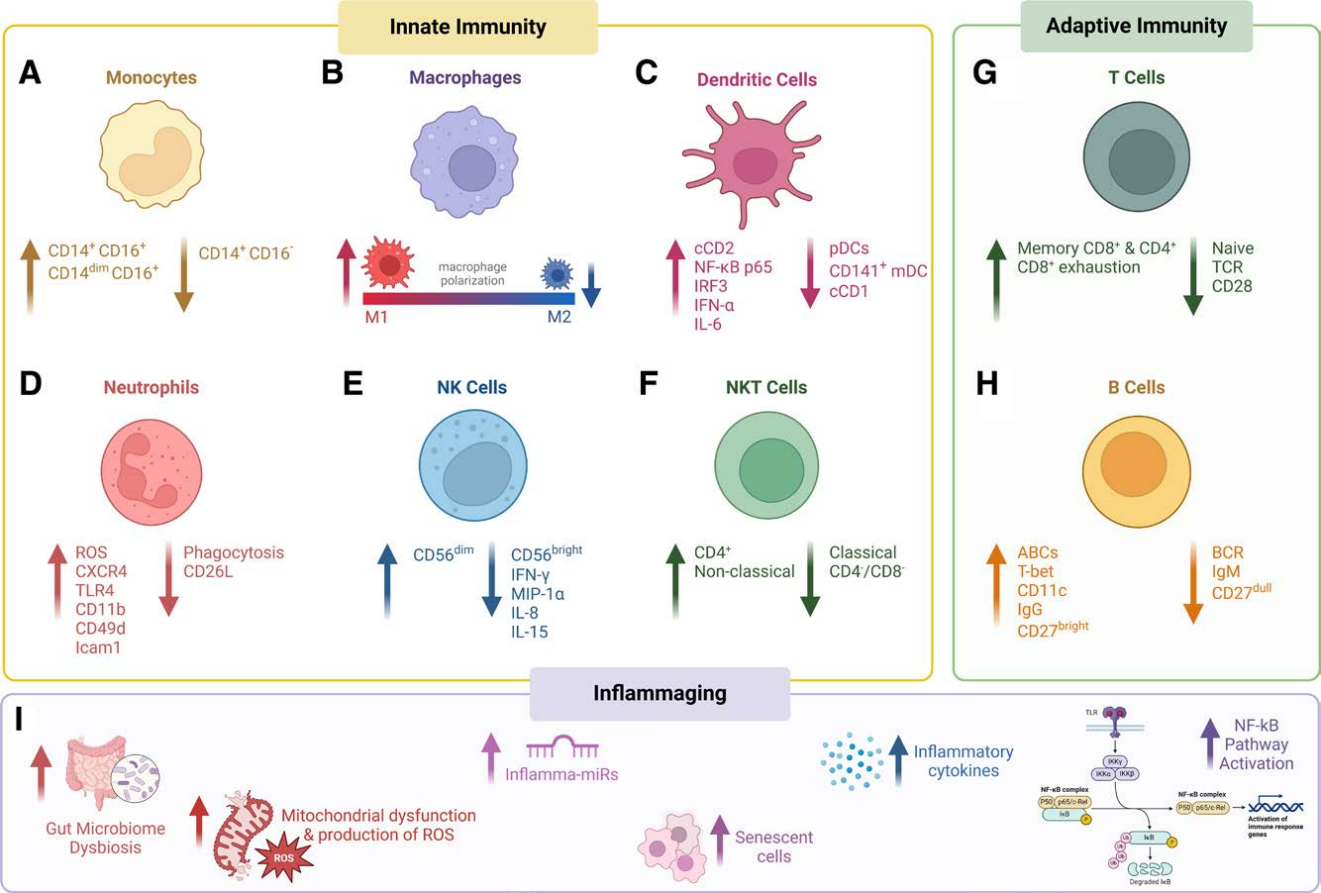
**Innate and adaptive aging immunity and inflammaging.** (A–F) Innate immune system; (G–H) Adaptive immune system; (I) Inflammaging.

Monocytes are crucial in regulating inflammation. Monocytes circulate in the bloodstream and are recruited to sites of infection and inflammation, where they can differentiate into macrophages or dendritic cells ^[11]^. Additionally, monocytes exhibit phagocytic activity and secrete pro-inflammatory and anti-inflammatory cytokines ^[12,13]^. Human monocytes can be broadly characterized into three main phenotypes: classical (CD14^+^CD16^−^), intermediate (CD14^+^CD16^+^), and nonclassical (CD14^low^CD16^+^) ^[14]^. Research has shown that older adults exhibit a decreased proportion of classical monocytes, and an increased proportion of intermediate and nonclassical monocytes compared with young and middle-aged adults ^[15]^. This shift in monocyte subtypes with age suggests an enhancement of cells with increased production of pro-inflammatory cytokines, promoting a state of chronic inflammation and an overall reduced ability to resolve inflammation in response to tissue damage or inflammatory insults ^[16]^. Despite these changes in monocyte proportion, the total number of monocytes is reported to remain relatively stable with age in humans ^[17]^ (Figure 1A).

### 2.2 Macrophages

Macrophages are primarily derived from monocytes that migrate from the bloodstream into tissues or are derived from progenitor cells in the yolk sac ^[18,19]^. While both types of macrophages have been shown to play important roles in the immune response, monocyte-derived macrophages are a major contributor in immune responses to infection or tissue damage, whereas yolk sac-derived tissue-resident macrophages are considered more important for tissue development and homeostasis maintenance ^[20]^. Macrophages can be broadly classified as either classically activated, pro-inflammatory (M1), or alternatively activated, anti-inflammatory (M2), although macrophages in tissues present as a spectrum with numerous intermediate subtypes ^[21]^. M1 macrophages are known for their strong antimicrobial ability, antitumor activity, and high capacity for antigen presentation ^[22,23]^. M1 macrophages are induced by T helper 1 (T_H_1)-derived cytokines, including interferon-gamma (IFN-γ), tumor necrosis factor-alpha (TNF-α), and granulocyte-macrophage colony-stimulating factor, as well as specific microbial-derived stimuli such as lipopolysaccharide (LPS), and secrete pro-inflammatory cytokines such as interleukin (IL)-1β, IL-6, IL-12, IL-23, and TNF-α ^[24]^. Conversely, M2 macrophages contribute to T helper 2 (T_H_2) immune responses, tissue regeneration, tumor proliferation, parasite clearance, and possess immunoregulatory functions ^[25]^. M2 macrophages are induced by T_H_2-derived cytokines, including IL-4 and IL-13 ^[24]^. Macrophages express toll-like receptors (TLRs) that enable them to detect pathogens with similar molecular patterns, activate inflammatory responses, and modulate overall immunity ^[26]^. TLRs also present foreign antigens via class I and II major histocompatibility complexes (MHCs) to T cells, thus facilitating antigen recognition ^[27]^.

In aging, macrophages shift toward a pro-inflammatory phenotype, characterized by enhanced M1 polarization and reduced M2 polarization, contributing to increased pro-inflammatory cytokine production ^[28,29]^. Aging also leads to altered TLR expression and decreased phagocytosis ^[30,31]^. It is also known that macrophages from older adults produce less TNF-α and IL-6 due to an age-related defect in the TLR1/2 pathway, impacting their ability to effectively recruit neutrophils and other immune cells ^[32]^. Additionally, studies in mice show that aged macrophages exhibit impaired phagocytosis, reduced reactive oxygen species (ROS) production, and diminished MHC class II expression following IFN-γ stimulation ^[33]^ (Figure 1B).

### 2.3 Dendritic cells

Dendritic cells (DCs) serve as a distinct class of antigen-presenting cells in the immune system that activate naive CD4^+^ and CD8^+^ T cells ^[34]^. They express high levels of the molecules required for antigen presentation, including MHC II, CD80, and CD86 ^[35,36]^, making them highly effective in triggering immune responses. DCs can be broadly classified into two main types: plasmacytoid DCs (pDCs) and myeloid DCs (mDCs). Myeloid DCs, also referred to as conventional DCs (cDCs), are further divided into two subsets: cDC1 correlating to CD141^+^ DCs, and cDC2 correlating to CD1c^+^ DCs. Both subsets are present in the circulation of humans ^[37]^. The cDC1 subsets are specialized in presenting antigens to CD8^+^ T cells and are crucial for antiviral and antitumor responses, whereas the cDC2 subset is involved in presenting antigens to CD4^+^ T cells and orchestrating helper T cell responses ^[38]^.

Aging impacts the composition and function of these DC subsets. One major alteration is a shift in hematopoiesis, which favors myeloid lineage cells such as cDCs, over lymphoid lineage cells such as pDCs ^[39,40]^. This shift results in a decrease in the number of circulating pDCs in the aged DC cell population ^[41–43]^, while the number of cDCs remains largely unchanged ^[41,43–45]^. Within the cDC population, however, the CD141^+^ subset declines in older individuals, whereas CD1c^+^ cDC numbers remain unaffected ^[46]^. Correspondingly, aging in rodents is associated with a decline in cDC1 cells and an increase in cDC2 cells ^[47–49]^.

Aging alters the responsiveness of DCs to TLR stimulation and cytokine production. In one study, pDCs and cDCs from elderly women demonstrated reduced TLR7/8-mediated stimulation compared with those from young, adult women ^[50]^. Further, in aged mice, cDCs are less effective in promoting the activation of naive CD8^+^ T lymphocytes and upregulating other stimulatory molecules ^[51]^. With respect to cytokine production, aging is associated with increased secretion of pro-inflammatory cytokines and decreased secretion of anti-inflammatory and immune-regulatory cytokines from DCs ^[52]^. This shift contributes to a diminished ability of DCs to present antigens to the adaptive immune system ^[47]^. Additionally, aged DCs exhibit enhanced basal activity of nuclear factor kappa B (NF-κB) p65, leading to heightened activation of the transcription factor IRF3, which in turn results in increased production of IFN-α and IL-6, promoting inflammation ^[53]^ (Figure 1C).

### 2.4 Neutrophils

Neutrophils are another cell type in the innate immune system critically involved in the immune response through activities such as phagocytosis and the release of ROS, which enhance their capacity for defense against infections. With aging, there is a notable increase in the prevalence of neutrophils within lymphoid tissues, including the bone marrow, lymph nodes, and spleen, which is closely associated with chronic inflammation ^[54,55]^.

Aging negatively affects neutrophil function, reducing their phagocytic capabilities ^[56]^ while increasing ROS production ^[57]^. This reduction in phagocytosis may be attributable to the impaired formation of neutrophil extracellular traps, which are crucial for capturing and neutralizing pathogens to prevent their spread ^[58–61]^. Furthermore, aging leads to changes in neutrophil surface marker expression, specifically increasing CXCR4, TLR4, CD11b, CD49d, and Icam1, and decreasing CD26L, which collectively shift neutrophils toward heightened inflammation, as well as impaired recruitment and functional responsiveness ^[62]^ (Figure 1D).

### 2.5 Natural killer cells

Natural killer (NK) cells are recognized as the principal lymphocytes that provide primary defense against cells infected by viruses or undergoing viral transformation ^[63,64]^. NK cells are essential for the elimination of senescent cells ^[65]^, resolution of inflammation ^[66,67]^, and induction of the adaptive immune response ^[68,69]^. NK cells are identified as CD56^+^ and CD3^−^, and are further categorized based on the cell surface expression of CD56 into CD56^bright^ and CD56^dim^ subsets, each possessing distinct functional properties ^[70]^. CD56^bright^ NK cells are primarily involved in cytokine production and immune regulation, while CD56^dim^ NK cells are more cytotoxic and are responsible for directly killing infected or transformed cells.

Both the quantity and composition of NK cells change with age. Specifically, there is a rise in the total number of NK cells, with an increase in CD56^dim^ and a decrease in CD56^bright^ populations ^[71–74]^. These changes correspond to a shift in the functional capacity of NK cells, impacting their effectiveness in immune responses. The reduction in CD56^bright^ cells, which are primarily responsive to cytokine challenges, may be another reason for decreased cytokine responsiveness with age ^[75–77]^.

One study revealed that the amount of IFN-γ, macrophage inflammatory protein (MIP)-1α, and IL-8 (but not TNF-α) produced by target cell-stimulated NK cells from older subjects was significantly lower than the levels secreted by NK cells from younger subjects ^[78]^. This reduced cytokine production can diminish the capacity of NK cells to orchestrate an effective immune response. Furthermore, with aging, the level of IL-15 decreases, which is important for the growth and maintenance of NK cells, promoting a decline in NK cell functionality ^[79]^ (Figure 1E).

Natural killer T (NKT) cells are a unique subset of T lymphocytes that exhibit functional characteristics of both typical T cells and NK cells ^[80]^. NKT cells are primarily categorized as CD1d-restricted classical NKT cells and non-CD1d-restricted nonclassical NKT cells. The frequency of CD1d-restricted classical NKT cells is significantly decreased in the peripheral blood of the elderly compared with young individuals. Further, within the CD1d-restricted classical NKT cells, there is an increase in the proportion of CD4^+^ cells and a decrease in the proportion of CD4/CD8 double-negative cells ^[81]^. Conversely, studies indicate that circulating levels of non-CD1d restricted nonclassical NKT cells increase with aging ^[82]^ (Figure 1F).

## 3. The adaptive immune system in aging

After the initial defensive response by the innate immune system, the adaptive immune system is activated to provide a more specific and long-lasting response against the pathogenic invasion. This system involves the activation and proliferation of T cells and B cells, which recognize antigens presented by antigen-presenting cells and mount a targeted immune response to destroy infected cells and neutralize and clear pathogens. In aging, many aspects of the adaptive immune system are compromised. In this section, we highlight the impact of aging on the adaptive immune system, specifically focusing on the known effects on T cells and B cells in human and rodent models.

### 3.1 T cells

T cells are essential components of the adaptive immune system and are involved in cell-mediated immunity. Mature T cells are characterized into various subsets, including helper T cells (CD4^+^), which assist other immune cells, and cytotoxic T cells (CD8^+^), which directly kill infected or cancerous cells. A critical aspect of T cell development is the establishment of a diverse T-cell receptor (TCR) repertoire, which enables the immune system to respond to a wide array of antigens.

The thymus gland is a crucial primary lymphoid organ where immature T cells develop and differentiate into mature naïve T cells ^[83,84]^. The thymus gland is large and active in childhood but undergoes thymic involution in aging. During this process, thymic tissue is replaced by adipose tissue, leading to a reduction in naïve T cells ^[85]^. This thymic involution leads to decreased diversity of peripheral T cells, a reduction in the TCR repertoire, and impaired immune function ^[86–88]^. Thymic involution is also linked to elevated levels of IL-1β, primarily produced by thymic macrophages, which suppresses thymic lymphopoiesis and T cell expansion ^[89]^.

In newborns, all naive T cells express CD28, a crucial co-stimulator for T cell activation and survival ^[90]^. However, as T cells undergo antigen-mediated activation and differentiation, they progressively lose CD28 expression ^[91]^. An increased proportion of CD28^−^ T cells in aging correlates with a decline in immune function ^[91]^. In particular, the CD8^+^CD28^-^ T cell subset is significantly increase in older individuals, making it a potential target for anti-aging interventions targeting immunosenescence ^[90,92]^. These cells are characterized by reduced immune responsiveness and shortened telomeres, and they are commonly linked to replicative senescence in aged humans ^[93]^ (Figure 1G).

Concomitant with the decrease in naive T cells with aging, there is a significant increase in the number of memory CD4^+^ and CD8^+^ T cells with pronounced expansion of CD8^+^ T cells ^[92,94]^. However, aging additionally promotes the accumulation of senescent and exhausted CD8^+^ T cells, representing up to 60% of all CD8^+^ T cells. Senescent CD8^+^ T cells exhibit cell cycle arrest, poor proliferation, and enhanced secretion of pro-inflammatory cytokines TNF-α, IL-18, and IL-29. Exhausted CD8^+^ T cells arise due to persistent antigen stimulation and exhibit similar abnormalities to senescent CD8^+^ T cells, including poor effector function and proliferation, which further contributes to the age-related decline in immune responses ^[95]^.

### 3.2 B cells

B cells, a crucial component of the adaptive immune system, are known for producing antibodies and presenting antigens to T cells. As individuals age, the number of B cells remains relatively stable, but the turnover rate of these cells declines, leading to decreased B cell function ^[96]^. Aging notably impacts the generation of pre-B cells in the bone marrow and mature peripheral B cells, resulting in a decelerated turnover of the peripheral B cell pool ^[97]^. For example, aged mice maintain a sustained or slightly reduced number of peripheral B cells in response to environmental antigens ^[98,99]^. However, aging is associated with a decreased diversity of naive B cells and peripheral B cell receptor sequences ^[100]^.

One significant subset of B cells affected by aging is age-associated B cells (ABCs). ABCs are identified by the expression of markers such as T-bet, CD11c, CD11b, and the absence of CD21. Studies have shown that ABCs increase in prevalence during aging ^[101,102]^. T-bet, a transcription factor belonging to the T-box family, is predominantly expressed in T cells and is crucial in differentiating naive CD4^+^ T cells into T_H_1 cells. T-bet additionally serves as a primary regulator of ABCs ^[103]^. Stimulation of ABCs with TLR agonists can lead to the secretion of autoantibodies, and ABCs enhance antigen presentation to T cells by increasing CD11c expression ^[102–106]^. T-bet levels increase across all memory B cell subgroups during aging, even without stimulation, further increasing CD11c expression in all B cell subsets following TLR7 activation ^[107]^. These changes contribute to the elderly population’s difficulties in mounting appropriate responses to new infections and developing immunity to novel antigens, which can be attributed to an increased abundance of IgG and a decreased level of IgM ^[108]^.

Memory B cells (MBCs) are another important aspect of the B cell lineage and can be identified by the expression of the CD27 marker, distinguishing two distinct populations: CD27^dull^ and CD27^bright^ MBCs ^[109]^. With aging, the ratio between CD27^dull^ and CD27^bright^ MBCs decreases with age ^[109,110]^. There is a notable decline in the CD27^dull^ MBCs, which play a pivotal role in bridging innate and adaptive immune functions, both in terms of their percentage and absolute number ^[110]^. Conversely, the number of CD27^bright^ MBCs increases, which could influence immune responses against familiar antigens ^[110]^. However, this increase may also compromise the overall capacity to respond to new infections, thus highlighting the complex and changes in B cell subset composition and function in aging (Figure 1H).

## 4. Inflammaging

A hallmark of aging is the development of low-grade chronic inflammation in tissues, known as inflammaging ^[111]^. This process is influenced by various factors, including oxidative stress, DNA damage, an increase in senescent cells, central obesity, impaired endocrine signaling, and microbial dysbiosis ^[112]^. Inflammaging involves both the downregulation of cells in the innate immune system and the upregulation of pro-inflammatory cytokines, such as C-reactive protein (CRP), IL-6, IL-18, and TNF-α ^[113,114]^. One of the central mechanisms driving inflammaging is the activation of the master inflammatory NF-κB signaling pathway, which acts as a central regulator of inflammation and immune response ^[115]^.

Another feature of aging is the accumulation of senescent cells (SCs) in various tissues, including: adipose, heart, lungs, arteries, kidneys, skin, and bones. SCs exhibit a hypersecretory phenotype termed senescence-associated secretory phenotype (SASP) ^[116–124]^. SCs secrete a range of pro-inflammatory cytokines and other factors, such as microvesicles, exosomes, microRNAs (miRs), non-coding RNAs, mitochondrial DNA fragments, nucleotides, and ROS ^[125–128]^. Notably, the expression of inflamma-miRs, such as miR-21 and miR-146a, increases during aging in response to tissue damage caused by chronic inflammation and cellular senescence ^[129]^. Collectively, SASP promotes inflammatory responses ^[126,127,130]^ and contributes to multiple chronic conditions associated with inflammaging and age-related diseases ^[131]^.

Mitochondria, the powerhouses of cells, additionally play a significant role in the aging process. In senescent cells, impaired and damaged mitochondria contribute to inflammaging and a pro-inflammatory state ^[132,133]^. Age-associated mitochondrial dysfunction is primarily characterized by mutations in the mitochondrial DNA (mtDNA). mTDNA is crucial for maintaining and regulating mitochondrial functions, however, mtDNA is highly susceptible to damage from replication cycle defects and ROS, especially given its limited repair mechanisms ^[134]^. Increased mtDNA mutations can impair the mitochondrial respiratory chain, heightening the levels of mitochondrial ROS (mtROS), which further contributes to cellular senescence and exacerbates inflammaging ^[135]^. Circulating mtDNA levels increase with age, potentially due to ejection from granulocytes (including eosinophils and neutrophils), endothelial cells, cell death (pyroptosis), and/or mtDNA replicative regulation. These mitochondrial changes in senescent cells significantly contribute to the maintenance of low-grade, chronic inflammation and elevated inflammatory cytokines, such as TNF-α, IL-6, and IL-1Ra, observed in aged individuals ^[136]^ (Figure 1I).

## 5. Changes of gut microbiota during aging

Numerous internal and external factors, including the living environment, physical activity, host genotype, breeding condition, antibiotic exposure, age, and dietary habits, significantly impact the composition and functionality of the gut microbiome ^[137–139]^. Aging results in substantial changes in the composition, function, and diversity of the gut microbiome ^[140]^. Specifically, the abundance of critical symbiotic organisms such as *Bacteroides*, *Bifidobacterium*, and *Firmicutes (Lactobacilli*) have been shown to decrease with age. In contrast, the prevalence of *Proteobacteria* (*Escherichia*) and other opportunistic microorganisms, such as *Fusobacterium*, *Parabacteroides,* and Ruminococcaceae increased with age ^[141–143]^. The abundance of short-chain fatty acid (SCFA)-producing bacteria, such as *Clostridium leptum* and *Eubacterium rectale,* also decreased with age, affecting immunological function ^[144]^.

Studies have revealed that the transplantation of microbiota from aged mice into young mice promotes intestinal inflammation, increases the translocation of bacterial components into the circulation, and stimulates T cells in the systemic compartment, leading to an increase of *Proteobacteria* in young transplanted mice ^[145]^. Importantly, SCFA supplements and transplantation of SCFA-producing bacteria protect rodents from aging-related disorders. For instance, transplantation of butyrate-producing bacteria in aged mice increases blood butyrate levels, reduces inflammation, and enhances insulin sensitivity ^[146]^.

## 6. Effects of diets on the immune system and gut microbiota in aging

### 6.1 High-fiber diet

A high-fiber diet provides numerous benefits for the immune system and gut microbiome during aging. However, dietary fiber intake among elderly individuals is approximately 40% lower, on average, than the recommended adequate intake (21 g/day for females; 30 g/day for males) ^[147,148]^. This section will discuss the known effects of high-fiber diets on the immune system, inflammation, and gut microbiota in aging.

Dietary fiber, particularly soluble fibers such as inulin, are fermented by gut bacteria into SCFAs such as butyrate. In aged mice, supplementation with inulin was found to decrease levels of the chemoattractant CXCL11, although it did not significantly alter pro-inflammatory cytokines IL-6, IL-1α, and TNF-α ^[149]^. Furthermore, increased butyrate levels from a high-fiber diet have been shown to reduce the expression of pro-inflammatory cytokines in microglia, thereby improving neuroinflammation associated with aging in mice ^[150]^. In humans, the consumption of high-fiber cereals was associated with reduced levels of inflammatory markers, specifically CRP, a marker of acute-phase inflammation, and IL-1Ra, a marker of inflammasome activation ^[151]^. However, fiber from fruits and vegetables did not similarly attenuate these inflammatory markers ^[151]^, possibly due to differences in fiber types (soluble versus insoluble), the presence of other bioactive compounds, or processing methods that may alter fiber bioavailability ^[152]^. This highlights the necessity for further research to understand how different fiber sources and their associated compounds uniquely influence inflammation.

In addition to affecting inflammation, high-fiber diets have been shown to significantly alter gut microbiota composition in aging. In a study examining the gut microbiota of elderly Chinese individuals, the high-fiber diet group exhibited a lower abundance of *Bacteroidales* and *Lachnospiraceae*, and a higher abundance of *Ruminococcaceae* ^[153]^. Additionally, a study on inulin intake in individuals aged 55–80 years found that increased inulin consumption was associated with higher microbial diversity and a greater abundance of *Bifidobacterium*, *Alistipes shahii, Anaerostipes hadrus*, and *Parabacteroides distasonis* ^[154]^.

In aged mice, a diet with 2.5% inulin altered the gut microbiome by increasing *Bifidobacterium* and *Faecalibaculum* and enhancing butyrate production, though this diet reduced alpha diversity. Further, inulin intake did not reduce overall community compositional differences between age groups, affect systemic inflammation, or improve measures of gut physiology ^[155]^. Another mouse study showed that inulin intake decreased the *Firmicutes* to *Bacteroidetes* ratio and reduced populations of dysbiotic bacteria. Notably, inulin increased *Bifidobacterium* in older mice and *Bacteroides* abundance in an age-specific manner ^[149]^.

Lastly, in a humanized mouse model of aging, one study demonstrated that inulin modulated the gut microbiome and metabolites in a sex-dependent manner. Inulin increased the proportion of *Bacteroides*, *Blautia*, and glycine, and decreased *Eggerthella*, *Lactococcus*, *Streptococcus*, trimethylamine, 3-hydroxyisobutyrate, leucine, and methionine in both sexes ^[156]^. Inulin had a greater effect on bacterial composition in females than males. Specifically, in females, inulin decreased *Faecalibaculum*, *Lachnoclostridium*, *Schaedlerella*, and phenylalanine, and increased *Parasutterella*, *Phocaeicola*, *Lachnospiraceae*, *Barnesiella*, *Butyricimonas*, glycine, propionate, acetate, and glutamate. In males, inulin decreased *Enteroccaceae*, *Odoribacter*, bile acids, malonate, thymine, valine, acetoin, and ethanol, and increased *Dubosiella*, pyruvate, and glycine ^[156]^. These findings underscore the importance of researching dietary interventions in both sexes to best tailor personalized nutritional strategies.

### 6.2 High-protein diet

Studies of high-protein diets (HPDs) have shown both beneficial and detrimental effects on the immune system in aging, depending on the diet composition and health status. Research suggests that increased protein intake may reduce exercise-induced inflammation. For example, a study of 100 older women (aged 60–90) engaged in resistance training found that plasma IL-6 levels were reduced in women who had increased consumption of lean red meat vs women who exercised without the dietary intervention ^[157]^. However, in other studies, an HPD was associated with a decline in anti-inflammatory factors and an elevation of pro-inflammatory factors. Specifically, an HPD enriched with omega-3 fatty acids, in addition to an 8-week exercise intervention, demonstrated a reduction in anti-inflammatory markers IL-10 and IL-1Ra in elderly individuals ^[158]^. Notably, elderly men in this study exhibited decreased pro-inflammatory markers ^[158]^. Furthermore, combination of HPD and low-carbohydrate diet has been shown to be immunosuppressive in aged rats ^[159]^. Lastly, in aged mice, an HPD (whole protein) increased levels of LPS, IL-6, and IL-10, with hydrolyzed protein additionally raising TNF-α ^[160]^.

HPD has also been shown to negatively affect gut microbiota composition and the production of gut microbiota-derived metabolites. In older women, HPD was associated with a reduction in butyrate-producing bacteria such as *Roseburia* and *Anaerostipes,* which may contribute to impaired gut barrier function ^[161,162]^. In HPD-fed rats, fecal acetate levels were significantly increased, likely due to the fermentation of undigested proteins by SCFA-producing bacteria in the large intestine ^[163,164]^. Additionally, elevated trimethylamine *In recent decades, dietary patterns among older adults have undergone a dramatic shift. In particular, there has been an overall reduction in fiber intake (described in Section 6.1) and an increase in the proportion of energy intake from fat*-oxide (TMAO), a gut microbiota-derived metabolite linked to increased cardiovascular disease risk ^[165,166]^, was observed in older men consuming high levels of protein (>1.6 g/kg body weight/day), but this effect was not observed in older women ^[167]^. In mice, HPD (whole protein) damaged the structure of the small intestine, reduced the number of goblet cells, and increased the abundance of *Streptococcus* and *Peptococcus*, while decreasing *Bifidobacterium*. However, hydrolyzed protein mitigated these adverse effects, improving small intestine structure, increasing goblet cell numbers, increasing *Bifidobacterium*, and reducing pathogenic bacteria ^[160]^ (Table 1). Other studies suggest that the quality, quantity, timing, and nitrogen availability of dietary protein consumption, as well as the intake levels of other nutrients, influence the gut microbial composition ^[175–177]^. Thus, more research is necessary to fully understand the complex interactions between dietary protein intake, immune function, and gut microbiota. While consumption of HPDs has become popular in recent years, these data further support the current dietary guidelines, including limiting protein intake to 1–1.2 g/kg body weight/day, while ensuring sufficient carbohydrate consumption ^[178]^.

**Table 1.** The effect of diet on microbiota and immune system components of aged mice and humans.

Diet	Model	Microbiota modifications	Immunomodulation
High-fiber	Aged humans	↑ *Ruminococcaceae* ^[153]^ ↑ *Microbial diversity* ^[154]^ ↑ *Bifidobacterium* ^[154]^ ↑ *Alistipes shahii* ^[154]^ ↑ *Anaerostipes hadrus* ^[154]^ ↑ *Parabacteroides distasonis* ^[154]^ ↓ *Bacteroidales* ^[153]^ ↓ *Lachnospiraceae* ^[153]^	↓ CRP ^[151]^ ↓ IL-1Ra ^[151]^
	Aged mice	↑ *Bifidobacterium* ^[149,155]^ ↑ *Faecalibaculum* ^[155]^ ↑ *Bacteroides* ^[149,156]^ ↑ *Blautia* ^[156]^ ↑ Glycine ^[156]^ ↓ Alpha diversity ^[155]^ ↓ *Firmicutes* to *Bacteroidetes* ratio ^[149]^ ↓ *Eggerthella* ^[156]^ ↓ *Lactococcus* ^[156]^ ↓ *Streptococcus* ^[156]^ ↓ Trimethylamine ^[156]^ ↓ 3-hydroxyisobutyrate ^[156]^ ↓ Leucine ^[156]^ ↓ Methionine ^[156]^	=/↓ Pro-inflammatory cytokines ^[149,150]^ ↓ CXCL11 ^[149]^
High-protein	Aged humans	=/↑ TMAO (gut microbiota-derived metabolite) ^[166,167]^ ↓ *Roseburia* ^[161]^ ↓ *Anaerostipes* ^[161]^	↓ IL-6 ^[157]^ ↓ IL-10 ^[158]^ ↓ IL-1Ra ^[158]^ ↓ Pro-inflammatory cytokines ^[158]^
	Aged mice	↑ Fecal acetate ^[163]^ ↑ *Streptococcus* ^[160]^ ↑ *Peptococcus* ^[160]^ ↑ Damage to small intestine ^[160]^ ↑/↓ *Bifidobacterium* ^[160]^ ↓ Goblet cells ^[160]^	↑ Immunosuppression ^[159]^ ↑ LPS ^[160]^ ↑ IL-6 ^[160]^ ↑ TNF-α ^[160]^ ↑ IL-10 ^[160]^
High-fat	Aged humans	–	–
	Aged mice	↑ *Firmicutes* ^[168]^ ↑ *Enterobacteriaceae* translocation to visceral organs ^[169]^ ↑ *Erysipelotrichaeceae* ^[170]^ ↑ *Lactobacillaceae* ^[170]^ ↑ *Lachnospiraceae* ^[170]^ ↑ *Bacteroidaceae* ^[170]^ ↑ *Bifidobacteriaceae* ^[170]^ ↓ *Ruminococcaceae* ^[171]^ ↓ *E. coli* ^[169]^	↑ M1 macrophage polarization ^[172]^ ↑ TNF-α ^[172,173]^ ↑ MCP-1 ^[172,173]^ ↑ TLR4 ^[173]^ ↑/↓ IL-1β ^[173,174]^ ↑ IL-6 ^[173]^ ↓ IgM ^[174]^

CRP, C-reactive protein; CXCL11, C-X-C motif chemokine ligand 11; IgM, Immunoglobulin M; IL, interleukin; IL-1Ra, interleukin 1 receptor antagonist; LPS, lipopolysaccharide; MCP-1, monocyte chemoattractant protein-1; TLR, toll-like receptor; TMAO, trimethylamine N-oxide; TNF, tumor necrosis factor.

### 6.3 High-fat diet

In recent decades, dietary patterns among older adults have undergone a dramatic shift. In particular, there has been an overall reduction in fiber intake (described in Section 6.1) and an increase in the proportion of energy intake from fat ^[179,180]^. Preclinical studies indicate that consuming a high-fat diet (HFD; 40–60% of calories from fat) affects both immune responses and gut microbiota profiles ^[181–184]^. This suggests that changes in dietary fat consumption could exacerbate age-related immune decline, contributing to overall immune dysfunction in aging.

In an aged mouse model, consumption of an HFD (vs a low-fat diet) significantly increased levels of the pro-inflammatory cytokines TNF-α and monocyte chemoattractant protein-1 (MCP-1) in white adipose tissue, irrespective of age. Both adipose tissue and liver exhibited pronounced M1 macrophage phenotypes, with a significant increase in adipose tissue NOS2 (an M1 macrophage marker) only in the aged mice fed an HFD ^[172]^. Additional rodent studies have associated HFD consumption with increased levels of other proinflammatory cytokines, such as IL-1β and IL-6, in both plasma and tissues ^[183–189]^. In aged mice with liver fibrosis, pro-inflammatory cytokines, which act as hepatic inflammatory mediators, show a strong response to HFD ^[173]^. This suggests that aging may increase susceptibility to hepatic fibrosis and inflammation under HFD ^[173]^. Further studies report that aged rodents fed HFD exhibit increased pro-inflammatory responses and M1 macrophage polarization, contributing to the development of steatohepatitis, higher TLR4 expression, and reduced levels of immunoglobulins, such as IgM ^[172,173,190,191]^.

HFD has also been shown to alter gut microbiota profiles in aging. As mice age, there is a gradual increase in the abundance of *Firmicutes* in those fed an HFD ^[168]^. The relative abundance of the *Firmicutes* phylum positively correlates with pro-inflammatory cytokines and has an inverse relationship with the tight junction protein claudin-1 ^[174]^. A decrease in claudin-1 weakens the gut barrier, contributing to its deterioration during aging. Additionally, HFD significantly reduces the abundance of *Ruminococcaceae* bacteria, responsible for butyrate production, in the gastrointestinal tracts of mice ^[171]^. Recent studies have shown that HFD-feeding in aged mice reduces the abundance of *E. coli* in the gut and promotes *Enterobacteriaceae* translocation to visceral organs, promoting dysbiosis ^[169]^. In other studies, bacteria from the families *Erysipelotrichaceae, Lactobacillaceae, Lachnospiraceae, Bacteroidaceae,* and *Bifidobacteriaceae* were overrepresented in aged HFD-fed mice ^[170]^.

Dietary fatty acids (FAs) are considered important regulators of inflammation. Specifically, omega-6 polyunsaturated fatty acids (PUFAs) have pro-inflammatory capacities, while omega-3 PUFAs have anti-inflammatory effects ^[192]^. Research has further elucidated that older adults who consume high amounts of dietary monounsaturated fatty acids exhibit elevated levels of pro-inflammatory cytokines such as IL-12 and TNF-α, whereas PUFA intake is negatively correlated with IL-12 levels ^[193]^.

## 7. Effects of dietary components on the immune system and gut microbiota in aging

### 7.1 Tryptophan

Tryptophan is a crucial dietary element that plays a prominent role in various physiological processes, including stress responses, mental well-being, oxidative stress, inflammatory responses, and gut health ^[194]^. There are several dietary sources of tryptophan, including: oats, bananas, dried prunes, milk, tuna fish, cheese, bread, chicken, turkey, peanuts, and chocolate ^[195]^.

**Figure 2.**
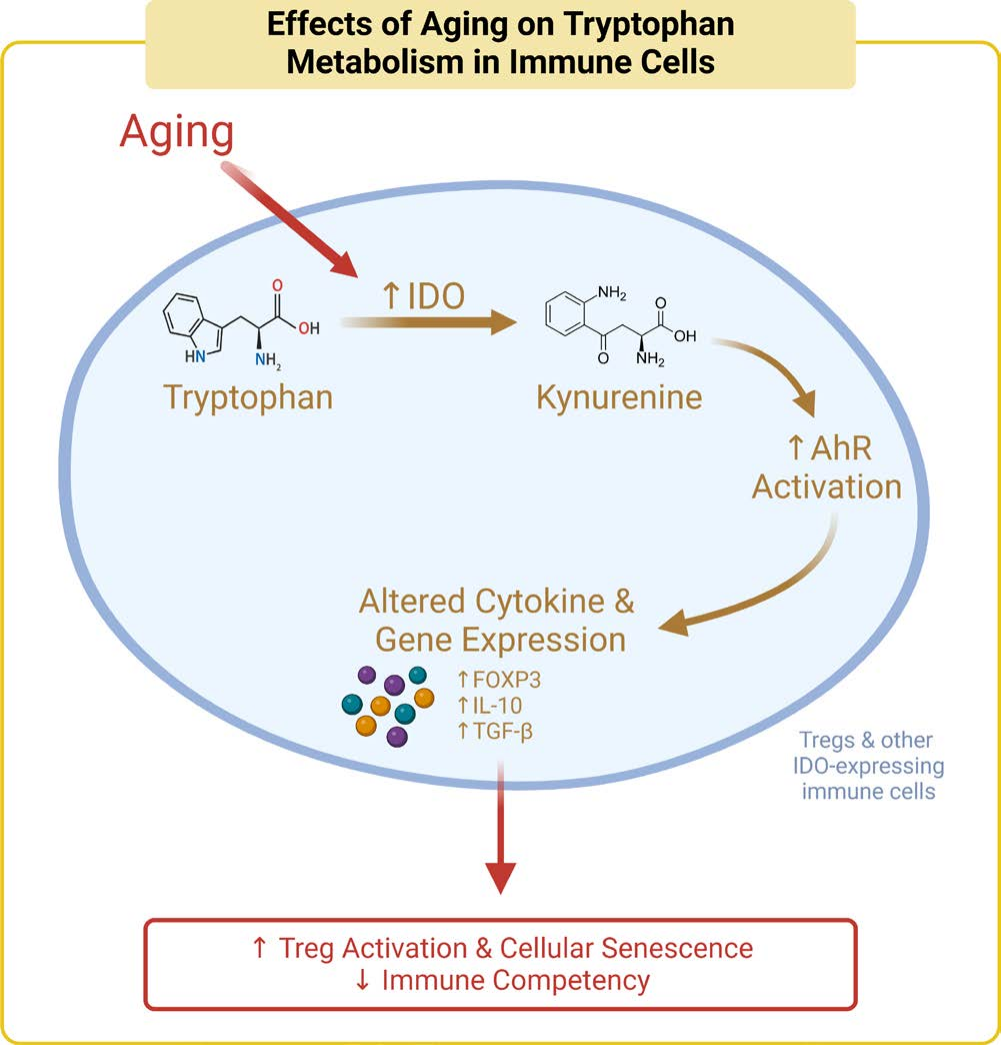
**The effects of aging on tryptophan metabolism in immune cells.** Aging increases activity of the rate-limiting tryptophan catalytic enzyme indoleamine-2,3-dioxygenase (IDO), which activates kynurenine and downstream aryl hydrocarbon receptor (AHR). Through FOXP3, IL-10, and TGF-β, this signaling cascade increases Treg activation, promotes cellular senescence, and decreases immune competency.

Tryptophan regulates the immune system through the kynurenine pathway, which degrades more than 95% of free tryptophan ^[196]^ via indoleamine-2,3-dioxygenase (IDO) to produce kynurenine ^[197–199]^ and intermediate metabolites like kynurenic acid and quinolinic acid, which in turn regulate immune functions and contributes to nicotinamide adenine dinucleotide (NAD)+ production ^[200]^ (Figure 2). Molecular mechanisms that regulate NAD+ during aging have been reviewed elsewhere ^[201]^. Thus, here, we have specifically focused on the role of tryptophan in the kyneurine pathway. During aging, increased levels of pro-inflammatory cytokines elevate IDO activity, resulting in an increased kynurenine/tryptophan ratio and activation of aryl hydrocarbon receptor (AhR) ^[202]^. Activation of AhR can induce immunosuppressive factors such as IL-10, Foxp3, and Treg ^[203–206]^, promoting thymus involution and immunosenescence ^[207,208]^. We have shown that aging is associated with decreased levels of tryptophan and its associated metabolite indole in mice during aging ^[209]^. A study in aged mice revealed that a tryptophan-deficient diet led to significant increases in IL-6, IL-17A, and IL-1α, decreased IL-27 levels, and changes in gut microbial composition compared with normal levels of tryptophan supplementation (0.2%) ^[194]^. Specifically, in tryptophan-deficient mice, there were alterations in the abundance of the Coriobacteriia class, *Acetatifactor* genus, Lachnospiraceae family, *Enterococcus faecalis* species, *Clostridium sp* genus, and *Oscillibacter* genus ^[194]^. Another study demonstrated that moderate tryptophan intake (0.4% of diet) decreased the relative abundances of *Erysipelatoclostridium, Enterococcus*, and *Dubosiella* while increasing the abundance of *Akkermansia*, which is associated with reduced oxidative stress, as well as SCFA-producing bacteria such as *Butyricimonas* and *Odoribacter* in aged mice ^[210]^. Conversely, higher tryptophan consumption (0.8% of diet) aggravated the gut mucosal barrier, oxidative stress, and inflammation, indicating that excessive tryptophan may induce a gut microbiota disorder in the aging process ^[210]^. It is also of note that other essential amino acids, such as methionine and branched-chain amino acids (BCAAs), are important for microbial metabolism, but their effects on aging immunity are currently understudied.

### 7.2 Omega-3 fatty acids

Omega-3 fatty acids are an important type of PUFA primarily obtained from fish, seafood, nuts, seeds, plant oils, eggs, and dairy products ^[211]^. Despite their nutritional importance, the elderly population often consumes suboptimal amounts of omega-3 fatty acids ^[212]^. Omega-3 fatty acids possess anti-inflammatory properties, as evidenced by studies showing that omega-3 deficiency in rats leads to elevated plasma levels of IL-6, CRP, and TNF-α ^[213]^. Similarly, in humans, omega-3 intake counteracts many obesity-related inflammatory and metabolic changes by inducing gut microbiome alterations that reduce pro-inflammatory circulating LPS, NF-κB activity, and inflammatory cytokine production ^[214]^.

Specifically in postmenopausal women, six months of omega-3 supplementation has been associated with reductions in TNF-α, MCP-1, ^[215]^ and CRP ^[201]^. Our work has shown that while both omega-3 supplementation and a weight-loss intervention can individually reduce levels of pro-inflammatory adipokines, cytokines, and growth factors in peri/postmenopausal women with obesity, the combination of omega-3 supplementation with >10% body weight loss led to the greatest impact on these factors ^[201]^. α-linolenic acid (ALA) is an omega-3 fatty acid that is a precursor to two other omega-3 fatty acids: eicosapentaenoic acid (EPA) and docosahexaenoic acid (DHA) ^[216]^. These omega-3 fatty acids have been shown to reduce inflammation and maintain homeostasis of the immune system in rodent and human models ^[217,218]^. Specifically, the consumption of omega-3 fatty acids, including ALA, EPA, and DHA, as dietary supplements, reduced the concentrations of pro-inflammatory eicosanoids, including prostaglandin E2 (PGE2), leukotriene B4 (LTB4), and leukotriene C4 (LTC4), as well as cytokines (including IL-1β, MCP-1, TNF-α, and CRP), in aged rats with diet-induced dyslipidemia ^[213,219]^. Additionally, increasing the intake of gamma-linoleic acid, EPA, and DHA has been shown to decrease lymphocyte proliferation in older adults ^[220]^. In elderly individuals, a diet rich in omega-3 fatty acids reduces pro-inflammatory cytokine IL-1β and the mammalian target of rapamycin (mTOR) in the skeletal muscle ^[221]^. The levels of IL-6, IL-1Ra, TNF-α, and CRP negatively correlate with total omega-3 fatty acids in aging populations ^[222–224]^ (Table 2). Conversely, an elevated ratio of omega-6 to omega-3 fatty acids has been correlated with increased levels of TNF-α and IL-6 ^[227]^. Omega-3 fatty acids additionally decrease inflammation through effects on leukocyte activity, eicosanoid synthesis, and T cell development ^[228]^.

**Table 2:** The effect of dietary supplements on immune system components in aging.

Supplement	Outcome
Tryptophan	↑ IL-27 ^[194]^ ↓ IL-6 ^[193]^ ↓ IL-17A ^[193]^ ↓ IL-1α ^[193]^
Omega-3 fatty acids	↓ IL-6 ^[213]^ ↓ CRP ^[213]^ ↓ TNF-α ^[213]^ ↓ Pro-inflammatory eicosanoids ^[219]^ ↓ IL-1β ^[219,221]^ ↓ MCP-1 ^[215,219]^ ↓ TNF-α ^[215,219]^ ↓ CRP ^[201,219]^ ↓ Lymphocyte proliferation ^[220]^ ↓ mTOR ^[221]^
Galacto-oligosaccharides	↑ Phagocytosis ^[170,225]^ ↑ NK cell activity ^[225,226]^ ↑ IL-10 ^[225,226]^ ↑ IL-8 ^[225,226]^ ↑ CRP ^[225,226]^ ↑ Immune surveillance ^[170]^ ↑ Chemotaxis ^[170]^ =/↓ IL-6 ^[225,226]^ =/↓ TNF-α ^[225,226]^ ↓ IL-1β ^[225,226]^ ↓ ROS ^[170]^ ↓ Senescence ^[170]^

Omega-3 intake also influences the diversity and composition of gut microbiota ^[229]^. For example, omega-3 fatty acids have been shown to promote microbiota diversity, increase *Bifidobacteria*, and reduce *Enterobacteria* in aged rodents, contributing to improvements in gut barrier function and intestinal permeability ^[184,230]^. ALA supplementation in aged mice also elevates SCFA acetate production and decreases TMAO generation ^[231]^. In a study of middle-aged and elderly women, increased omega-3 fatty acid consumption was correlated with gut microbiota diversity, decreased inflammation, and greater abundance of the *Lachnospiraceae* family ^[232,233]^ (Table 3). Importantly, *Lachnospiraceae* plays a principal role in converting long-chain polysaccharides into SCFA, such as butyrate and propionate ^[234]^.

**Table 3:** **The impact of dietary components on the gut microbiota in aging.** The presence of tryptophan, omega-3 fatty acids, and galacto-oligosaccharides in the diet alter composition of the gut microbiome in aging.

Dietary component	Species
Tryptophan	↑ *Akkermansia* ^[210]^ ↑ *Butyricimonas* ^[210]^ ↑ *Oderibacter* ^[210]^ ↓ *Dubosielle* ^[210]^ ↓ *Enterococcus* ^[210]^ ↓ *Erysipelatoclostridium* ^[210]^
Omega-3 fatty acids	↑ *Bifidobacteria* ^[184,230]^ ↑ *Lachnospiraceae* ^[232]^ ↓ *Enterobacteria* ^[184,230]^
Galacto-Oligosaccharides (GOS)	↑ *Bifidobacterium* ^[225,226]^ ↑ *Lactobacillus-Enterococcus* ^[225]^ ↑ *C. Coccoides-E. rectale* ^[225]^ ↑ *Muribaculaceae* ^[170]^ ↑ *Prevotellaceae* ^[170]^ ↑ *Rikenellaceae* ^[170]^ ↑ *Oscillospiraceae* ^[170]^ ↑/↓ *Bacteriodes* ^[225,226]^ ↓ *C. histolyticum* ^[225]^ ↓ *E. coli* ^[225]^ ↓ *Desulfovibrio* ^[225]^

### 7.3 Galacto-oligosaccharides

Galacto-oligosaccharides (GOS) are prebiotics recognized for their health benefits, particularly in modulating the gut microbiota ^[235–237]^. Dietary sources of GOS include legumes such as lentils, chickpeas, and beans, as well as dairy products and certain root vegetables ^[238]^. Studies investigating Bimuno (B-GOS) supplementation, have observed immunomodulatory effects in elderly individuals. Specifically, studies demonstrated that B-GOS supplementation increased phagocytosis, NK cell activity, increased production of anti-inflammatory cytokines (IL-10), increased production of some pro-inflammatory cytokines (IL-8, CRP), and reduced production of other pro-inflammatory cytokines (IL-6, IL-1β, and TNF-α) ^[225,226]^ (Table 2). In the context of HFD and brain health during aging, one study demonstrated that a combination of GOS + fructooligosaccharide (FOS) supplementation reduced neuroinflammation, specifically restoring phagocytosis, surveillance, and chemotaxis, while also decreasing ROS and senescence ^[170]^.

Additionally, GOS supplementation has been shown to modulate gut microbiota. One study showed that B-GOS supplementation (5.5 g/d) significantly increased the abundance of both *Bifidobacterium* and *Bacteroides* ^[226]^ (Table 3). Another study suggested that B-GOS at the same dosage increased *Bifidobacterium*, *Lactobacillus-Enterococcus*, *C. Coccoides-E. rectale*, and decreased *Bacteroides*, *C. histolyticum*, *E. coli,* and *Desulfovibrio* compared with both baseline and placebo treatment ^[225]^. In this study, the numbers of species/groups that increased (*Bifidobacterium, Lactobacillus-Enterococcus, and C. Coccoides-E. rectale*) were positively correlated with NK cell activity and increased percentage of cells engaged in phagocytosis ^[225]^. Additionally, in the study investigating the effects of GOS+FOS supplementation on brain health during aging in the context of HFD, it was found that supplementing GOS+FOS preserved the populations of potentially anti-inflammatory bacteria *Muribaculaceae*, *Prevotellaceae*, *Rikenellaceae*, and *Oscillospiraceae* in HFD mice, whereas these were diminished in mice fed HFD alone ^[170]^. Lastly, some studies have suggested that GOS supplementation causes a reduction in overall bacterial diversity in mice, though this has not been consistently reported in humans ^[226,239–241]^.

## 8. Conclusion and future research

With the rapid increase of the aging population worldwide, it is crucial to develop effective strategies to combat age-related diseases to improve the quality of life for the elderly. A major deficiency of aging is the decline in immunological function, which makes older adults more vulnerable to infections, inflammatory diseases, and vaccine failure. Understanding how diet and specific dietary components affect immune function and gut microbiota can help to develop new strategies to promote healthy aging.

A high-fiber diet has been suggested as a promising intervention to combat immunosenescence and inflammaging by enhancing SCFA production, which promotes immune cell function and maintains gut barrier integrity. Conversely, evidence suggests that a high-fat diet exacerbates inflammation and negatively impacts gut microbiota, indicating the critical need for older adults to reduce fat intake. Moderate protein intake (1–1.2 g/kg) has been suggested to reduce inflammation when combined with resistance training, but excessive protein intake combined with low carbohydrate intake may increase inflammatory markers such as TMAO and promote immunosuppression. Therefore, balancing protein consumption with other macronutrients is essential to ensure its beneficial effects while minimizing potential adverse effects.

Omega-3 fatty acids and moderate tryptophan intake demonstrate notable anti-inflammatory effects, contributing to reduced cytokine production and improved gut microbiota diversity. GOS has been shown to exert anti-inflammatory effects and positively modulate gut microbiota by increasing beneficial bacteria, which enhance immune function and reduce inflammation.

In conclusion, maintaining a balanced diet, as well as incorporating dietary components including omega-3 fatty acids, tryptophan, and GOS can support healthy aging by alleviating inflammation, improving gut microbiota profiles, and overall immune functionality. Continued research of the interactions between diet, immune function, and gut microbiota will be critical to developing personalized nutritional strategies to improve health outcomes of the aging population, ultimately increasing healthspan.

## Conflicts of interest

The authors declare that they have no conflicts of interest.

## Funding

This work was supported by NIH grants: R01AG064869 and R01CA269726.

## Acknowledgments

All figures were created with BioRender.com.
